# Limits of “Skills And Drills” Interventions to Improving Obstetric and Newborn Emergency Response: What More Do We Need to Learn?

**DOI:** 10.9745/GHSP-D-16-00372

**Published:** 2016-12-23

**Authors:** Jim Ricca

**Affiliations:** a Jhpiego, Washington, DC, USA.

## Abstract

A “skills and drills” intervention in 4 hospitals in Karnataka, India, produced modest improvement in provider knowledge and skills but not in actual response to obstetric and newborn emergencies. We explore possible explanations, which include (1) the need for a more intensive intervention; (2) other weaknesses in the health system; and (3) behavioral or organizational barriers related to hierarchical structures, roles, and team formation.

See related article by Varghese.

In this issue of GHSP, Varghese and colleagues present the results of a set of "skills and drills" activities aimed at improving maternal and newborn emergency care in a high-mortality setting in Karnataka, India.^1^ The Argentine Institute for Clinical Effectiveness and Health Policy with funding from the Maternal Health Task Force provided catalytic training and orientation activities over 2.5 days to a core group of coordinators. These coordinators formed a regional team composed of 8 nurses and 20 physicians from medical colleges in Karnataka. They instituted the skills and drills practice-improvement activities in 4 intervention facilities with mainly local human and material resources. The activities consisted of a 2-day skills course at the study facilities followed by visits to the facilities every 2 months over a 12-month period to carry out drills and supportive supervision activities.

Practitioners deemed the skills and drills intervention feasible and acceptable, and there was a modest increase in provider knowledge and skills. But the intervention appeared not to have achieved its desired results in improvements in clinical practice, either in the identification of cases in need of emergency intervention or in improved response to identified emergencies. This may be attributable to continued poor reporting, but since that also was a focus of the intervention, this lack of improvement in documentation is itself instructive.

## PREVIOUS RELATED WORK

Teamwork and emergency preparedness have been shown to be key determinants of successful obstetric emergency response.^2^ Therefore, in addition to coordinating the technical aspects of care, “skills and drills” interventions, including realistic simulation exercises for obstetric and neonatal emergencies, have been used for several decades in a variety of clinical settings in high-income countries to improve the quality of providers' responses as they work as a team in stressful and time-sensitive situations.^3^^–^^6^ Such simulation approaches focus on procedures for preparation and coordinated clinical response. Facilitators set a scripted realistic emergency scenario, and providers respond as they would in a real situation. The simulation is often videotaped, and the clinical team debriefs afterward to review and reflect on what could have been done differently to improve the response.

A low-technology obstetric emergency simulation-based course named Advance Life Support in Obstetrics has been in use in North America since 1993.^7^ Over the last 10–15 years, use of obstetric simulation in high-technology simulation centers has become more common. There has been increasing interest in the use of simulation approaches in low- and middle-income countries as well, usually done on-site in a facility rather than in a special simulation center. For example, PRONTO International has introduced simulation-based training in 7 countries, much of it focusing on improving obstetric emergency care.^8^ In addition, the Expanding Maternal and Newborn Survival (EMAS) Program, a bilateral maternal newborn care project in Indonesia supported by the U.S. Agency for International Development, introduced emergency preparedness and simulation approaches at moderate scale.^9^

There is evidence that such simulation approaches can improve provider knowledge, skills, and confidence, but current evidence is thin that these approaches improve provider practices, especially when implemented at larger scale.^10^^,^^11^ A current Cochrane protocol seeks to explore this gap in knowledge (or possibly enlighten us on the extent to which this knowledge gap has been addressed).^12^ It is interesting that even in high-income settings, there is interest in implementing simulations as they are usually done in low- and middle-income countries—that is, as more realistic on-site simulations instead of being conducted in a simulation center—because there is some indication that this may give better results in terms of system-oriented improvements.^13^

Simulation approaches can improve provider knowledge, skills, and confidence, but current evidence is thin that they improve provider practices.

## POSSIBLE REASONS WHY THE INTERVENTION IN INDIA HAD LIMITED EFFECT

There are several possible explanations for the negative results reported by Varghese et al. First, the authors suggest, as a possible explanation for the lack of practice improvement, that the intervention did not address other systems weaknesses (beyond provider skills and team functioning) such as commodity stock-outs or human resources shortages and turnover. The presence of a provider with knowledge and skills is a necessary but not a sufficient condition for the delivery of quality care. If a skilled provider does not possess a needed commodity, then clearly she will not be able to perform a lifesaving practice that depends on that commodity. Although the authors assert that this may account for the lack of improved care, they do not present data to show how much of a role systems weaknesses such as commodity supply might have played. However, the fact that the diagnosis of reported complications was not better than the comparison facilities suggests that commodity issues do not fully account for the negative results, as diagnosis and reporting requires nothing more than provider knowledge, skills, and motivation. So this underreporting of complications remains unexplained. Providers told the mentoring teams that they only report emergencies when something is done. This leaves open an important set of questions. Are providers still not recognizing problems and so not intervening? Or are they recognizing problems but still not thinking them serious enough to intervene? Or did providers intervene in some cases but did not report them because of poor outcomes?

Lack of improvement in provider practices in the India intervention may be due to other systems weaknesses, such as commodity stock-outs.

We should also consider other possible explanations for the negative results, related to provider motivation and behavior. This may have been a well-designed and suitable intervention, but there simply might not have been enough of it. As an intervention to change provider behavior and encourage teamwork, this was a relatively "light-touch" intervention. Supervisory and simulation visits happened only once every 2 months, were externally driven, and were not reinforced by more frequent on-site, peer-to-peer mentoring. So it is possible that this intervention was on the right track, but the dose was too low to have the desired effect. Emergencies happen infrequently, are high-risk situations, and require rapid responses. In order to build confidence, the desired behavior may simply need to be modeled and practiced more often to truly cement it.

The simulation intervention in India may have been on the right track, but the “dose” may have been too low to have the desired effect.

Another possible explanation for the negative results could be that the intervention did not effectively address important barriers to teamwork. How improvement goals are set has been shown to be critical.^14^ Teams also function best in situations where there is a flat, non-hierarchical organizational structure.^2^ In a hierarchical health system, there may be hesitancy on the part of some providers to form, lead, or play an equal role on a team. Were there procedures and protocols for determining who would call for the formation of a team response and then lead it? Could a nurse lead a team in which a physician was a member, or play an equal role, for instance? Did lack of clarity about roles cause critical delays in emergency response? Providers may have been able to play non-traditional roles during simulations, but in actual practice they might well have reverted to usual behavior even when this may have blunted the effectiveness of the emergency response. In their debriefs, the teams noted the quality of communication and rapport, but it is not clear how much the training and mentoring directly addressed possible organizational/behavioral barriers to team formation and functioning nor to the differentiation of roles within the team. In addition to modeling the desired behavior of team members during drills, it may be necessary to discuss with providers how team members ought to act, how this might be different from usual practice, and their reactions to this.

## IMPLICATIONS FOR FUTURE EFFORTS

The authors have given us an example of an intervention designed to address the need for clinical practice improvement for obstetric and newborn emergencies. The failure to show an effect on this primary outcome opens up a set of questions that future investigations ought to address. Future investigations should systematically take account of other systems issues in order to better establish what incremental value there may be to a skills and drills intervention in terms of clinical practice improvement. But other questions also need to be answered, such as the “dose” of the activities needed to improve team formation and functioning in emergencies. At a minimum, it would be instructive for future investigations to “open the black box” of what actually is happening on the ward after the skills and drills training to see how teams are functioning in emergency situations and how, if at all, this has been influenced by the skills and drills initiative. Further formative inquiry is also warranted on barriers and facilitators to team formation and functioning, centering on the issues of hierarchy, role differentiation, and team communication. The issues identified are likely to be quite context-sensitive, with the consequence that those interested in skills and drills interventions will need to engage in implementation research on proposed solutions in their own environment. Finally, of course, we need to remain open to the idea that there may be other significant behavioral barriers to effective provider response to emergencies not addressed directly by skills and drills (e.g., fear of negative consequences for unsuccessful intervention, exacerbated by a perceived non-supportive and punitive organizational environment). Future investigators may also be able to address identified problems through “restructuring the incentive environment,”^15^ perhaps rewarding desired behavior through one or more mechanisms (e.g., peer recognition, financial incentives). To be effective, such restructuring of the organizational environment would require the inclusion of facility managers and health system leaders in the design at a level above that of individual providers and facilities.

Future simulation interventions should focus on barriers and facilitators to team formation and functioning, including issues of hierarchy, role differentiation, and team communication.

## Notes

**First Published Online:** 2016 Dec 19

## References

[1] Varghese B Krishnamurthy J Correia B Panigrahi R Washington M Ponnuswamy V Limited effectiveness of a skills and drills intervention to improve emergency obstetric and newborn care in Karnataka, India: a proof-of-concept study. Glob Health Sci Pract. 2016;4(4):582–593. 10.9745/GHSP-D-16-00143 PMC5199176 27993924

[2] Cornthwaite K Alvarez M Siassakos D Team training for safer birth. Best Pract Res Clin Obstet Gynaecol. 2015;29(8):1044–1057. 10.1016/j.bpobgyn.2015.03.020. 25979351

[3] Clark EA Fisher J Arafeh J Druzin M Team training/simulation. Clin Obstet Gynecol. 2010;53(1):265–277. 10.1097/GRF.0b013e3181cc4595. 20142662

[4] Bergh AM Baloyi S Pattinson RC What is the impact of multi-professional emergency obstetric and neonatal care training? Best Pract Res Clin Obstet Gynaecol. 2015;29(8):1028–1043. 10.1016/j.bpobgyn.2015.03.017. 25937554

[5] Rovamo L Nurmi E Mattila MM Suominen P Silvennoinen M Effect of a simulation-based workshop on multidisplinary teamwork of newborn emergencies: an intervention study. BMC Res Notes. 2015;8:671. 10.1186/s13104-015-1654-2. 26563963 PMC4643499

[6] Reime M Johnsgaard T Kvam FI Aarflot M Breivik M Engeberg JM Simulated settings; powerful arenas for learning patient safety practices and facilitating transference to clinical practice. A mixed method study. Nurse Educ Pract. 2016;21:75–82. 10.1016/j.nepr.2016.10.003. 27769018

[7] American Academy of Family Practice (AAFP) [Internet]. Leawood (KS): AAFP; c2016. Advanced Life Support in Obstetrics (ALSO); [cited 2016 Nov 9]. Available from: http://www.aafp.org/cme/programs/also.html

[8] Pronto International [Internet]. Seattle (WA): Pronto International; C 2016 [cited 2016 Oct 22]. Available from: http://prontointernational.org/

[9] Wasisto B Budiharsana M Koblinsky M Bartlett A Report of the mid‐term evaluation–Expanding Maternal and Newborn Survival (EMAS) program USAID/Indonesia. [Jakarta (Indonesia): U.S. Agency for International Development; 2014]. Available from: http://pdf.usaid.gov/pdf_docs/PA00JZ5Q.pdf

[10] Crofts J Winter C Sowter M Practical simulation training for maternity care–where we are and where next. BJOG. 2011;118 Suppl 3:11–16. 10.1111/j.1471-0528.2011.03175.x. 22039887

[11] Cox T Seymour N Stefanidis D Moving the needle: simulation's impact on patient outcomes. Surg Clin North Am. 2015;95(4):827–838. 10.1016/j.suc.2015.03.005. 26210974

[12] Fransen AF Banga FR van de Ven J Mol BJ Oei S Multi-professional simulation-based team training in obstetric emergencies for improving patient outcomes and trainees' performance (Cochrane protocol). London: The Cochrane Collaboration; 2015 Available from: http://www.cochrane.org/CD011545/PREG_multi-professional-simulation-based-team-training-in-obstetric-emergencies-for-improving-patient-outcomes-and-trainees-performance 10.1002/14651858.CD011545.pub2 PMC8094450 33325570

[13] Petrosoniak A Auerbach M Wong A Hicks C In situ simulation in emergency medicine: moving beyond the simulation lab. Emerg Med Australas. 2016 Oct 17. Epub ahead of print. 10.1111/1742-6723.12705. 27748042

[14] Gardner A Kosemund M Hogg D Heymann A Martinez J Setting goals, not just roles: improving teamwork through goal-focused debriefing. Am J Surg. 2016;pii:S0002–9610(16) 30652-3. Epub ahead of print. 10.1016/j.amjsurg.2016.09.040. 27765182

[15] Janssen W Ngirabega Jde D Matungwa M Van Bastelaere S Improving quality through performance-based financing in district hospitals in Rwanda between 2006 and 2010: a 5-year experience. Trop Doct. 2015;45(1):27–35. 10.1177/0049475514554481. 25406257

